# Laminin heparin-binding peptides bind to several growth factors and enhance diabetic wound healing

**DOI:** 10.1038/s41467-018-04525-w

**Published:** 2018-06-04

**Authors:** Jun Ishihara, Ako Ishihara, Kazuto Fukunaga, Koichi Sasaki, Michael J. V. White, Priscilla S. Briquez, Jeffrey A. Hubbell

**Affiliations:** 1 Institute for Molecular Engineering, University of Chicago, Chicago, IL 60637 USA; 2 Present Address: Department of Bioengineering, Tokyo Institute of Technology, 226-8501 Yokohama, Kanagawa Japan; 3 Present Address: Department of Applied Chemistry, Faculty of Engineering, Kyushu University, Nishi-ku, Fukuoka 819-0395 Japan

## Abstract

Laminin, as a key component of the basement membrane extracellular matrix (ECM), regulates tissue morphogenesis. Here, we show that multiple laminin isoforms promiscuously bind to growth factors (GFs) with high affinity, through their heparin-binding domains (HBDs) located in the α chain laminin-type G (LG) domains. These domains also bind to syndecan cell-surface receptors, promoting attachment of fibroblasts and endothelial cells. We explore the application of these multifunctional laminin HBDs in wound healing in the type-2 diabetic mouse. We demonstrate that covalent incorporation of laminin HBDs into fibrin matrices improves retention of GFs and significantly enhances the efficacy of vascular endothelial cell growth factor (VEGF-A165) and platelet-derived growth factor (PDGF-BB) in promoting wound healing in vivo, under conditions where the GFs alone in fibrin are inefficacious. This laminin HBD peptide may be clinically useful by improving biomaterial matrices as both GF reservoirs and cell scaffolds, leading to effective tissue regeneration.

## Abstract (web summary)

Laminins are important regulators of epidermal wound healing. Here, the authors show that laminins bind to multiple growth factors via their heparin-binding domains, and that incorporation of these domains into fibrin matrices increases growth factor retention, promoting wound healing in type 2 diabetic mouse models.

## Introduction

Laminins are the most abundant glycoproteins of the basement membrane extracellular matrix (ECM) and can be found in almost all tissues of the body. They play essential roles in the establishment of tissue architecture and stability, and provide cells with a structural scaffold. As such, laminins are involved in a variety of biological processes ranging from tissue survival, angiogenesis^1^, and neural development^2^, to skin reepithelialization and wound healing^3–5^, and even cancer metastasis^6,7^. Laminins have been shown to regulate core cellular activities, such as adhesion, apoptosis, proliferation, migration, and differentiation. Laminin is structured as a heterotrimer comprising three chains, α, β, and γ, that assemble into a cross shape^3,8,10^. At least 16 different isoforms of laminin exist, made by various combinations of the five α (LAMA1–5), three β (LAMB1–3), and three γ (LAMC1–3) chains that have been identified. Isoforms are accordingly named by their –αβγ chains: for instance, laminin-332 contains LAMA3, LAMB3, and LAMC2 chains. The differential expression of laminin isoforms depends on tissue type and state^7,11^.

In skin, for example, the epithelial basement membranes contain laminin-111 and laminin-211 during embryogenesis, but predominantly laminin-332 and laminin-511 in adults, the latter isoform expression seeming to diminish with age. Interestingly, dermal fibroblasts can transiently reexpress laminin-211 after wounding^12^. Moreover, dermal blood vessels specifically express laminin-511 and laminin-311, in addition to laminin-411 commonly found in endothelial basement membrane^3^.

In skin wound healing, laminin has been shown to play a critical role in reepithelialization and angiogenesis^1,3^. Indeed, LAMA possesses five laminin-type G domain (LG) modules at the C-terminus, arranged in a tandem array, that are differentially processed under homeostatic conditions or during tissue repair^9,15^. In fact, LAMA3, LAMA4, and LAMA5 chains are physiologically cleaved by proteases, such as plasmin and elastase, in the linker sequence between the LG3 and LG4 domains^13–16^, and processing in this region has been shown to correlate with the speed of wound closure^13^. As an example, laminin-332 is present in a cleaved form under homeostatic conditions; however, the expression of laminin-332 is upregulated after injury and the LG4 and LG5 domains are subsequently more present in wounds^13,17^. The release of LG4–LG5 module has been demonstrated to undergo further processing that releases bioactive peptides^13^, which promote blood vessel formation and keratinocyte migration, notably via syndecan binding^1,18–20^. In addition, laminin LG modules have been shown to bind to heparin sulfate, perlecan and fibulin-1^21^, as well as to a number of integrins, e.g., α3β1, α6β1, α7β1, and α6β4^22^.

In the past, ECM glycoproteins, including laminin, have been mainly considered for their biomechanical role in providing substrates for cell adhesion and migration, via direct interactions with cell-surface receptors^23^. Later, some ECM proteins have raised interest for their ability to regulate the partitioning and bioavailability of soluble signaling molecules within tissues, thus highlighting a new role for the ECM in coordinating the spatiotemporal release of these molecules. Examples of such soluble signals are growth factors (GFs), which are key morphogenetic proteins broadly involved in the control of core cellular behaviors, and which have been shown to be crucial for wound healing^24–26^. Particularly, fibronectin^27,28^, tenascin-C^29^, and fibrinogen^30^ have been reported to directly bind to GFs, controlling their release kinetics in vivo, the matrix acting as a GF reservoir. Importantly, it has been noticed that GF binding occurs at the heparin-binding domains (HBDs) of ECM glycoproteins, especially in fibronectin^27,28^, tenascin-C^29^, and fibrinogen^30^. Sustained release of GFs from the ECM eventually enhances and prolongs GF-receptor signaling. Furthermore, the proximity of GF-binding sites and integrin-binding sites in some ECM glycoprotein chains, as in fibronectin^28^ and tenascin-C^31^, can induce synergistic GF signaling by clustering GF receptors and integrins at the cell surface. In terms of laminin HBD, some of laminin HBDs have been identified in the LG domains^32–34^

Because laminin has a HBD, we hypothesized that GFs bind to laminin, as we had observed for other ECM proteins comprising a HBD^27–30^. Despite the importance of laminin in the ECM composition and its role in tissue morphogenesis, interactions between laminin and GFs have been poorly investigated to date. Here, we show direct interactions between laminin and several GFs through laminin HBDs. We then demonstrate the potential of laminin’s HBD to prolong the GF retention from fibrin matrices in vivo, leading to promotion of chronic wound healing, which may hold clinical translational potential.

## Results

### Multiple GFs bind to multiple isoforms of laminin

**Fig. 1:**
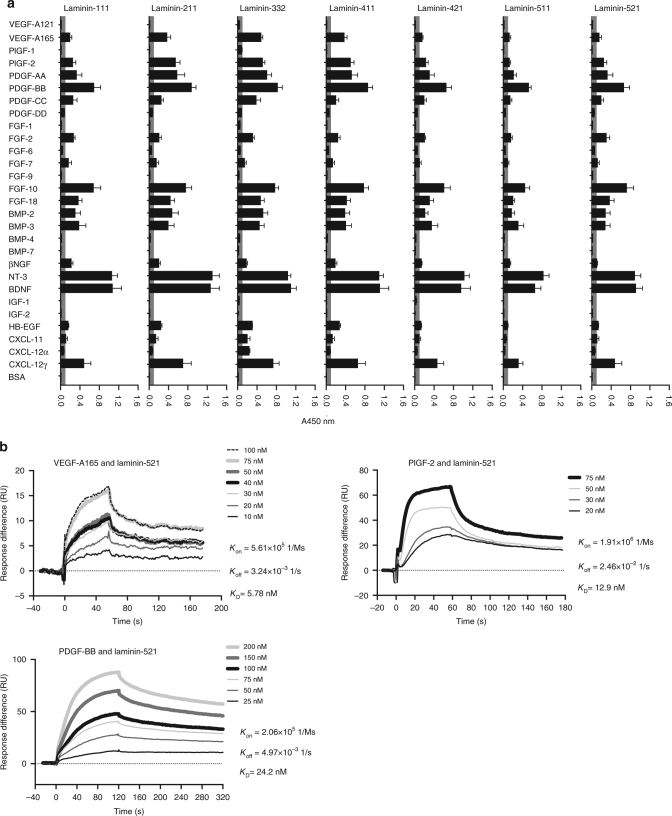
Laminin binds promiscuously to GFs and chemokines. **a** Binding of multiple isoforms of full-length laminin (–111, –211, –332, –411, –421, –511, and –521) to GFs and CXCL chemokines were measured by ELISA. A450 nm represents absorbance at 450 nm. BSA-coated wells served as negative controls (*n* = 4, mean ± SEM). Signals greater than 0.1 (gray box) are considered to be significant. **b** Affinities (*K*_D_ values are shown) of full-length laminin against VEGF-A165, PlGF-2, and PDGF-BB were measured by SPR. A SPR chip was functionalized with laminin-521 (~2000 resonance units (RU)), and each GF was flown over the chip at indicated concentrations. Curves represent the specific responses (in RU) to laminin obtained. Experimental curves were fitted with Langmuir binding kinetics. Binding kinetics values [dissociation constants (*K*_D_) and rate constants (*K*_on_ and *K*_off_)] determined from the fitted curves are shown. Two experimental replicates

We first examined the capacity of a variety of full-length laminin isoforms (–111, –211, –332, –411, –421, –511, and –521) to bind GFs from the vascular endothelial growth factor (VEGF)/platelet-derived growth factor (PDGF), fibroblast growth factor (FGF), bone morphogenetic protein (BMP), neurotrophin (NT), insulin-like growth factor (IGF), EGF, and CXCL chemokine families, for which we had previously observed binding to other ECM proteins, including fibronectin, vitronectin, tenascin-C, osteopontin, and fibrinogen^27,28,35^, as well as those that reportedly modulate wound healing^36^. Binding of laminin to absorbed GFs was detected using an antibody against laminin, and signals greater than 0.1 were considered to be indicative of a binding event. Overall, we found that multiple GFs strongly bound to all tested laminin isoforms (Fig. 1a). Specifically, from the VEGF/PDGF family, VEGF-A165, placental growth factor (PlGF)-2, PDGF-AA, PDGF-BB, and PDGF-CC bound to all isoforms of laminin, in contrast to VEGF-A121, PlGF-1, and PDGF-DD which did not show binding. From the FGF family, we observed that FGF-2, FGF-7, FGF-10, and FGF-18 bound to all laminin isoforms, whereas FGF-1, FGF-6, and FGF-9 did not. Among the BMPs, BMP-2 and BMP-3 showed binding to laminins, but not BMP-4 and BMP-7. NT-3 and brain-derived neurotrophic factor (BDNF) showed strong binding toward all tested laminin isoforms, while β-nerve growth factor (βNGF) bound only weakly. Neither IGF-1 nor IGF-2 displayed significant binding to laminins. In addition, heparin-binding epidermal growth factor (HB-EGF) weakly bound to laminins. As to the tested chemokines, C–X–C motif ligand (CXCL)-12γ bound to all laminin isoforms, whereas CXCL-11 and CXCL-12α bound weakly to laminin-332 but not to the other isoforms.

Next, we measured the affinities between laminin-521, as an example, and VEGF-A165, PlGF-2, and PDGF-BB using surface plasmon resonance (SPR). SPR chips were functionalized with laminin-521, and GFs were flown over the surface. The obtained binding curves were fitted with Langmuir binding kinetics to calculate specific dissociation constants (*K*_D_) (Fig. 1b). *K*_D_ values were 5.8 nM for VEGF-A165, 12.9 nM for PlGF-2, and 24.2 nM for PDGF-BB. The nM range of *K*_D_ values demonstrated the strong binding affinities of laminin-521 to the selected GFs.

### GFs bind to the HBDs of laminin

**Fig. 2:**
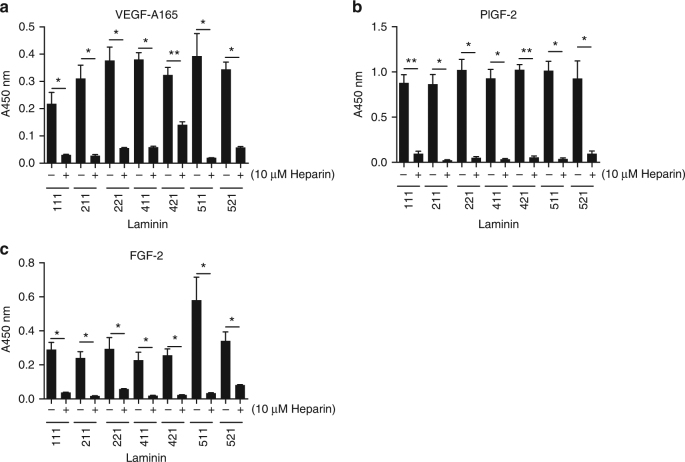
Excess heparin inhibits GF–laminin binding. Inhibition of GF-binding to laminin (–111, –211, –221, –411, –421, –511, and –521) by excess heparin. ELISA plates were coated with 10 µg/mL laminin and further incubated with 1 μg/mL **a** VEGF-A165, **b** PlGF-2, or **c** FGF-2 solution in the absence or presence of excess (10 μM) heparin. Bound GFs were detected using a specific antibody for each GF (*n* = 4, mean ± SEM). Statistical analyses were performed using the Mann–Whitney *U* test by comparing the signals with and without heparin. *p < 0.05, **p < 0.01. Two experimental replicates

**Fig. 3:**
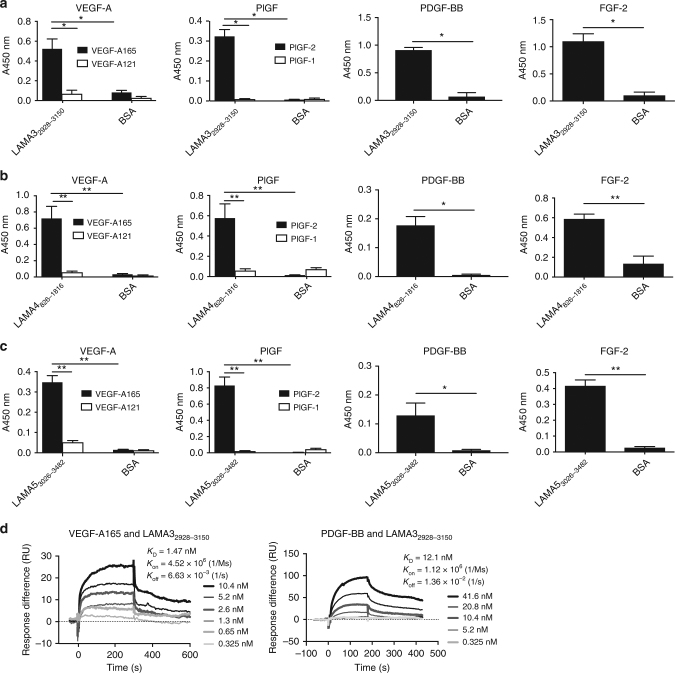
GFs bind to LG domain derived from LAMA3, LAMA4, and LAMA5. Affinity of GFs against recombinant laminin LG domains. ELISA plates were coated with 1 µg/mL **a** LAMA3_2928–3150_, **b** LAMA4_826–1816_, or **c** LAMA5_3026–3482_ and further incubated with 1 μg/mL of VEGF-A165, VEGF-A121, PlGF-2, PlGF-1, PDGF-BB, or FGF-2 solution. Bound GFs were detected using a specific antibody for each GF (*n* = 4, mean ± SEM). Statistical analyses were performed using the Mann–Whitney *U* test by comparing the signals obtained from the laminin domain- and the BSA-coated wells. *p < 0.05, **p < 0.01. **d** Affinities (*K*_D_ values are shown) of laminin LAMA3_2928–3150_ against VEGF-A165 and PDGF-BB were measured by SPR. A SPR chip was functionalized with the laminin LAMA3_2928–3150_ recombinant protein (~1000 RU), and each GF was flown over the chip at indicated concentrations. Curves represent the specific responses (in RU) to laminin. Experimental curves were fitted with Langmuir binding kinetics. Binding kinetics values [dissociation constants (*K*_D_) and rate constants (*K*_on_ and *K*_off_)] determined from the fitted curves are shown. Two experimental replicates

Because the GFs that bound to laminins have also been previously reported to bind to other ECM glycoproteins through HBDs^27,30,35^, we hypothesized that HBDs of laminins might be responsible for the interactions between GFs and laminin. To address this hypothesis, enzyme-linked immunosorbent assays (ELISA) were repeated for VEGF-A165, PlGF-2, or FGF-2 in the presence of heparin added in excess (10 µM). As a result, we observed that excess heparin inhibited GF binding to laminin (Fig. 2a–c), supporting that laminin HBDs mediated the interactions with GFs. To further confirm this, we tested direct GF binding to the LG domains from human LAMA3, LAMA4, and LAMA5, within which HBDs of laminin were localized^32^. We found that VEGF-A165, PlGF-2, PDGF-BB, and FGF-2 bound to laminin LG domains LAMA3_2928–3150_, LAMA4_826–1816_, and LAMA5_3026–3482_, in contrast to VEGF-A121 and PlGF-1 which did not show any binding (Fig. 3a–c), as tested by ELISA. The binding affinities between LAMA3_2928–3150_ and VEGF-A165 or PDGF-BB were then measured by SPR, and *K*_D_ values were 1.2 nM for VEGF-A165, and 10.2 nM for PDGF-BB (Fig. 3d). These data again demonstrated the strong affinities of the laminin LG domain to the tested GFs.

**Fig. 4:**
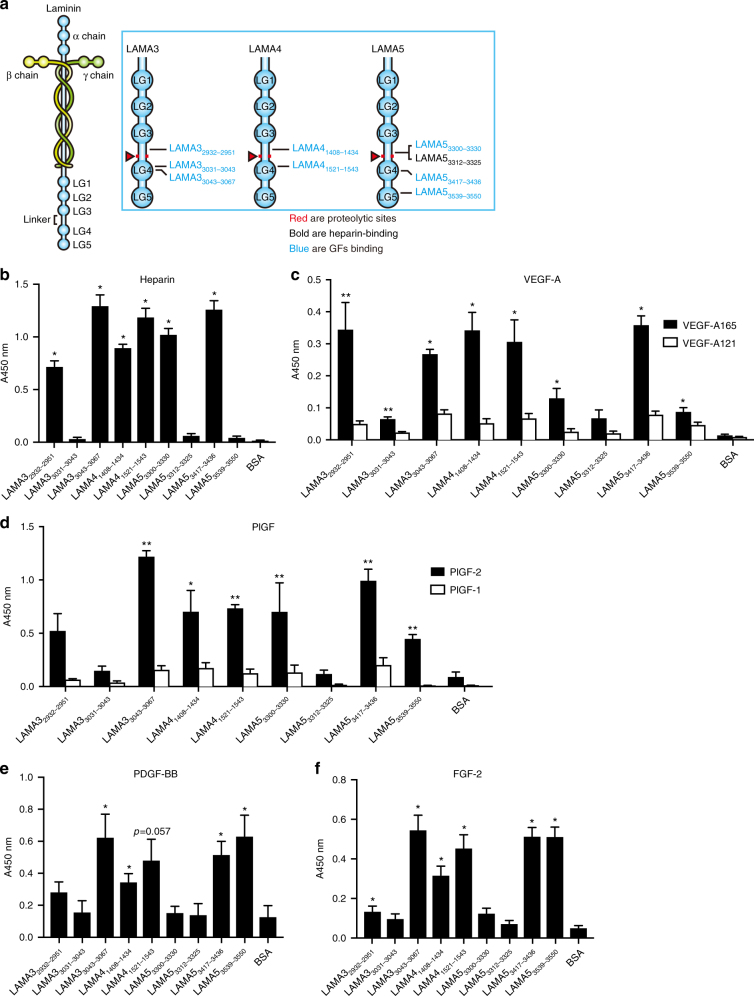
GFs bind to laminin HBD derived from LAMA3, LAMA4, and LAMA5. **a** The location of laminin-derived peptides in the LG domain of LAMA3, LAMA4, and LAMA5 chains. **b–f** Affinity of heparin and GFs against chemically synthesized peptides derived from the LG domain of LAMA3, LAMA4, and LAMA5 chains. ELISA plates were coated with 10 µg/mL laminin peptide and further incubated with **b** biotinylated heparin, **c** VEGF-A165 and VEGF-A121, **d** PlGF-2 and PlGF-1, **e** PDGF-BB, or **f** FGF-2. Concentrations were 1 μg/mL for GFs and 10 µg/mL for heparin. Bound heparin was detected with streptavidin, and bound GFs with a specific antibody for each GF (*n* = 4, mean ± SEM). Statistical analyses were performed using the Mann–Whitney *U* test by comparing the signals obtained from the laminin peptide- and the BSA-coated wells. *p < 0.05, **p < 0.01. Two experimental replicates

We next examined the binding of GFs to chemically synthesized laminin LG domain peptides, the sequences of which are all derived from human laminin sequences (Supplementary Table 1, Fig. 4a). These peptides are putative HBDs; they were determined based on previous reports with mouse or human HBD sequences^32^, or are positively charged sequences located within the linker domain between the LG3 and LG4 domains in LAMA3, LAMA4, and LAMA5. Of nine tested peptides, six bound to heparin (i.e., HBDs), namely LAMA3_2932–2951_, LAMA3_3043–3067_, LAMA4_1408–1434_, LAMA4_1521–1543_, LAMA5_3300–3330_, and LAMA5_3417–3436_ among which LAMA3_2932–2951_, LAMA4_1408–1434_, and LAMA5_3300–3330_ are derived from the LG3–LG4 linker. Interestingly, LAMA5_3312–3325_, which is a subdomain of LAMA5_3300–3330_, did not bind to heparin.

Finally, the affinities of VEGF-A, PlGF, PDGF-BB, and FGF-2 to these peptides were examined (Fig. 4b–f). We observed that all heparin-binding peptides showed significant binding to some GFs. Indeed, LAMA3_3043–3067_, LAMA4_1408–1434_, and LAMA5_3417–3436_ bound to VEGF-A165, PlGF-2, PDGF-BB, and FGF-2. LAMA4_1521–1543_ showed similar results except for binding to PDGF-BB, which was not statistically significant. LAMA3_2932–2951_ and LAMA5_3300–3330_ preferentially bound to VEGF-A165 and FGF-2, and VEGF-A165 and PlGF-2, respectively. As to the non-heparin-binding peptides, LAMA5_3312–3325_ did not show particular binding to any tested GF. Interestingly, LAMA5_3539–3550_, which did not show binding to heparin, significantly bound to all tested GFs, and LAMA3_3031–3043_ bound to VEGF-A165. None of the tested laminin-derived peptides bound to VEGF-A121 nor to PlGF-1, consistent with the results obtained in Figs. 1 and 3. To examine sequence specificity of this binding to GFs, we produced a scrambled sequence LAMA3_3043–3067_ peptide (Supplementary Fig. 1); scrambling the sequence of LAMA3_3043–3067_ decreased the binding signals between LAMA3_3043–3067_ and VEGF-A165, PlGF-2, PDGF-BB, and FGF-2, compared to its native form. Taken together, these data suggest that GFs bind to the HBDs of laminin, located in the LG3–LG4 linker or in LG4–LG5 domains.

### Laminin HBD peptides promote adhesion of cells

**Fig. 5:**
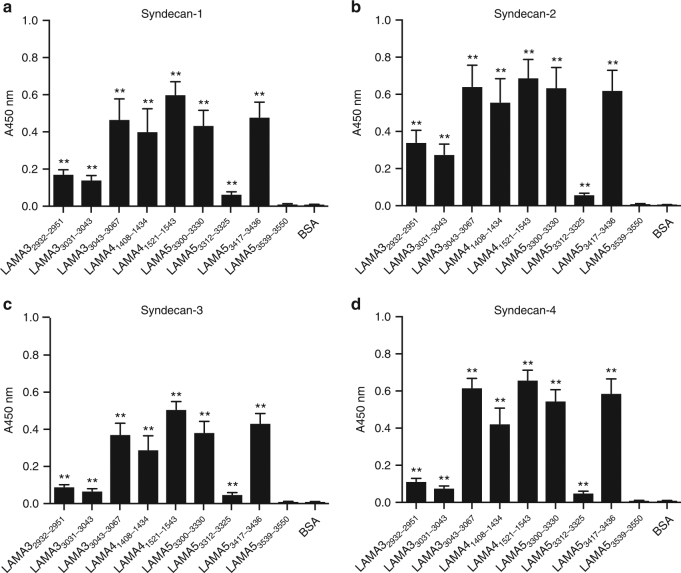
Syndecans bind to laminin HBD derived from LAMA3, LAMA4, and LAMA5. Affinity of syndecans to chemically synthesized peptides derived from the laminin LAMA3, LAMA4, and LAMA5 LG domains. ELISA plates were coated with 10 µg/mL laminin peptide and further incubated with 1 μg/mL of **a** syndecan-1, **b** syndecan-2, **c** syndecan-3, or **d** syndecan-4. Bound syndecans were detected using an antibody against histidine-tag on the recombinant syndecans (*n* = 8, mean ± SEM). Statistical analyses were performed using the Mann–Whitney *U* test by comparing the signals obtained from the laminin peptide- and the BSA-coated wells. *p < 0.05, **p < 0.01. Two experimental replicates

**Fig. 6:**
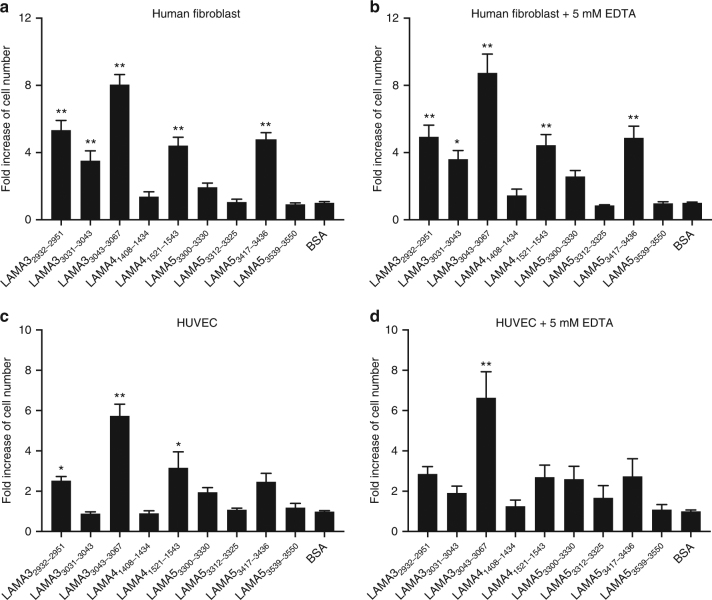
Laminin HBD peptides promote cell adhesion in vitro. **a**, **b** A total of 3000 cells/well human lung fibroblasts were cultured **a** without or **b** with 5 mM EDTA in FGM-2 culture media containing 1% FBS. **c**, **d** A total of 3000 cells/well HUVEC were cultured **c** without or **d** with 5 mM EDTA in EBM-2 culture media containing 100 ng/mL VEGF-A165 and 1% FBS. Cells were plated on 1 μg/mL laminin peptide pre-coated non-tissue culture-treated plates and incubated for 30 min at 37 °C. After plate washes, cell numbers were quantified using a CyQUANT assay (*n* = 10, mean ± SEM). The signals obtained from BSA-coated wells are normalized to 1, and relative fold increases of cell numbers were calculated. Statistical analyses were performed using ANOVA with Tukey’s test. Kruskal–Wallis test followed by Dunn’s multiple comparison was used in **b**, **c**. *p < 0.05, **p < 0.01. Two experimental replicates

Because the laminin HBDs have been reported to bind to syndecan^32^, a key cell-surface adhesion molecule, we tested syndecan binding to the synthesized laminin-derived peptides (Fig. 5a–d). LAMA3_3043–3067_, LAMA4_1521–1543_, LAMA4_1408–1434_, LAMA5_3417–3436_, and LAMA5_3300–3330_ showed significant binding to all isoforms of recombinant syndecans, i.e., syndecan 1–4. LAMA3_2932–2951_, LAMA3_3031–3043_, and LAMA5_3312–3325_ showed weak binding to the tested syndecans, while LAMA5_3539–3550_ did not show binding to any syndecan isoform. Because laminin-derived peptides that interact with syndecans may further promote cell adhesion by providing binding substrates, we tested fibroblasts and HUVEC adhesion to plates coated with these peptides. We indeed observed the enhancement of fibroblast attachment on LAMA3_2932–2951_, LAMA3_3031–3043_, LAMA3_3043–3067_, LAMA4_1521–1543_, and LAMA5_3417–3436_-coated surfaces (Fig. 6a). Fibroblast binding was observed even in the presence of ethylenediaminetetraacetic acid (EDTA), consistent with syndecan function (Fig. 6b). Of these peptides, LAMA3_2932–2951_, LAMA3_3043–3067_, and LAMA4_1521–1543_ also promoted HUVEC attachment (Fig. 6c), even in the presence of EDTA in the case of LAMA3_3043–3067_ (Fig. 6d). Interestingly, peptides that promoted both fibroblast and HUVEC adhesion in vitro through syndecan binding were those that we previously found to be laminin HBDs (Fig. 4a). VEGF-A165 increases the degree of migration of HUVEC cells in vitro (Supplementary Fig. 2). However, both in the presence and absence of VEGF-A165, LAMA3_3043–3067_ did not increase the degree of cell migration. A summary of laminin-derived peptide interactions is shown in Supplementary Table 2.

### Incorporation of laminin HBD prolonged GF retention

**Fig. 7:**
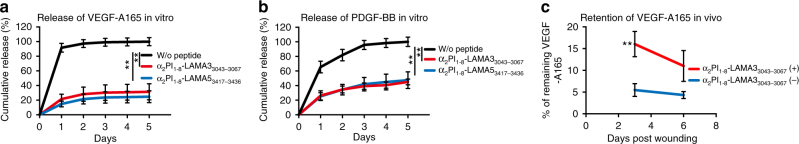
GF retention in fibrin matrices is enhanced by incorporating laminin HBD peptide. **a**, **b** GF retention in the fibrin matrix. α_2_PI_1–8_-LAMA3_3043–3067_ or α_2_PI_1–8_-LAMA5_3417–3436_ peptide-functionalized fibrin matrices were made in the presence of VEGF-A165 or PDGF-BB, and incubated in eight volumes of physiological buffer for 5 days. The buffer was changed each day, and released GFs were quantified daily. The graphs show the cumulative release of **a** VEGF-A165 or **b** PDGF-BB over 5 days (*n* = 4; mean ± SEM). All data points for laminin HBD peptides were statistically significant compared to controls without laminin HBD peptide (*p* < 0.01, Mann–Whitney *U* test). **c** Fibrin matrices containing VEGF-A165 (200 ng/wound) with or without α_2_PI_1–8_-LAMA3_3043–3067_ peptide were placed on the full-thickness back-skin wounds in 10–11-week-old C57BLKS/J-m/Lepr db (db/db) mice. After 3 and 6 days, retention of VEGF-A165 after 3 and 6 days in the fibrin matrix and the tissue surrounding the wound (2 mm beyond the wound margin) were quantified. Laminin HBD(+) day 3: *n* = 10, other groups: *n* = 8, mean ± SEM. Mann–Whitney *U* test; **p < 0.01. Two experimental replicates

We then sought to determine whether laminin HBD peptides, which showed binding to GFs, were able to improve the retention of VEGF-A165 and PDGF-BB within the fibrin matrix. Both VEGF-A165 and PDGF-BB are crucial factors for angiogenesis^37^. These GFs are known to be quickly released from fibrin matrices upon delivery, which limits their wound-healing efficacy in vivo^28,30,35^. For this purpose, we selected LAMA3_3043–3067_ and LAMA5_3417–3436_ laminin HBD peptides, and fused them to a transglutaminase-reactive sequence from the α_2_-plasmin inhibitor^28^ to allow their covalent incorporation by factor XIIIa into fibrin matrices during polymerization. GF release from fibrin matrices containing α_2_PI_1–8_-LAMA3_3043–3067_, α_2_PI_1–8_-LAMA5_3417–3436_, or no laminin-derived peptide was then monitored daily and quantified by ELISA (Fig. 7a, b). As expected, we observed that VEGF-A165 and PDGF-BB were quickly released from the fibrin matrix (>85% released after 24 h). However, incorporation of either α_2_PI_1–8_-LAMA3_3043–3067_ or α_2_PI_1–8_-LAMA5_3417–3436_ allowed significant retention of VEGF-A165 and PDGF-BB into matrices, which were respectively released after 5 days, for VEGF-A165 (α_2_PI_1–8_-LAMA3_3043–3067_: 25%, α_2_PI_1–8_-LAMA5_3417–3436_: 31%) and for PDGF-BB (α_2_PI_1–8_-LAMA3_3043–3067_: 45%, α_2_PI_1–8_-LAMA5_3417–3436_: 47%). These data highlight the key biological role of laminin in sequestering GFs into the ECM, and demonstrate the potential of laminin HBD peptides to control GF delivery from fibrin biomaterials (Fig. 7a, b). We next evaluated the effect of α_2_PI_1–8_-LAMA3_3043–3067_ on GF retention in diabetic wounds in the type-2 diabetic db/db mouse in vivo (Fig. 7c). Incorporation of α_2_PI_1–8_-LAMA3_3043–3067_ into fibrin matrices significantly enhanced the amount of VEGF-A165 remaining in the wounds 3 days after treatment, showing that incorporation of α_2_PI_1–8_-LAMA3_3043–3067_ prolongs retention of GFs in vivo.

### Laminin HBD potentiates GFs and promotes wound healing

**Fig. 8:**
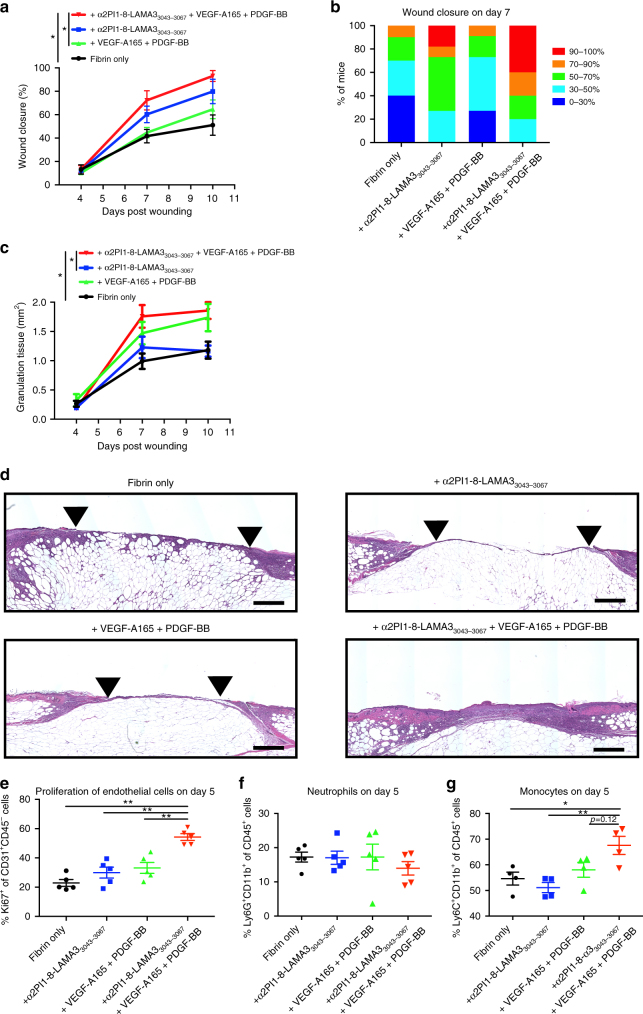
Delivering GFs and laminin HBD peptide enhances skin wound healing. Full-thickness back-skin wounds in 10– 11-week-old C57BLKS/J-m/Lepr db (db/db) mice were treated with combined VEGF-A165 (100 ng/wound) and PDGF-BB (50 ng/wound). Four groups were tested: fibrin only, fibrin functionalized with α_2_PI_1–8_-LAMA3_3043–3067_ peptide, fibrin containing admixed GFs, and fibrin functionalized with α_2_PI_1–8_-LAMA3_3043–3067_ peptide containing GFs. After 4, 7, and 10 days, **a**, **b** wound closure and **c** granulation tissue area were evaluated by histology (means ± SEM, day 4: *n* = 6, day 7: fibrin only, and α_2_PI_1–8_-LAMA3_3043–3067_ peptide + GFs, *n* = 10; other treatment groups, *n* = 11, day 10: α_2_PI_1–8_-LAMA3_3043–3067_ peptide, *n* = 8, α_2_PI_1–8_-LAMA3_3043–3067_ peptide + GFs, *n* = 9, and other treatment groups, *n* = 7). **b** The proportions of the mice were categorized by the degree of healing after day 7 of wound treatment. **d** Wound histology (hematoxylin and eosin staining) at day 7. Red arrows indicate tips of the epithelium tongue. The granulation tissue (pink–violet) is characterized by a large number of granulocytes with nuclei that stain in dark-violet or black. Muscle under the wounds is stained in red. Fat tissue appears as transparent bubbles. Scale bar = 800 µm. **e**–**g** A total of 5 days after the wound treatment, **e** proliferation of CD31^+^CD45^–^ endothelial cells is assessed by Ki67^+^ marker, and **f** the frequency of Ly6G^+^CD11b^+^ neutrophils within CD45^+^ cells and **g** the frequency of Ly6C^+^CD11b^+^ monocytes within CD45^+^ cells were determined using flow cytometry (means ± SEM). *P < 0.05, **P < 0.01, ANOVA with Tukey’s test. Two experimental replicates

Although the etiology of non-healing wounds is multifaceted in diabetes, the progression to a non-healing phenotype is related to poor blood vessel formation^38,39^. Thus, induction of mature blood vessels is a crucial step for diabetic wound-healing. Previous studies have reported a synergistic effect between angiogenesis inducers VEGF-A165 and PDGF-BB in wound healing^37^, and more precisely topical application of VEGF-A165 improves wound closure^40^ and PDGF-BB promotes the amount of granulation tissue in the type-2 diabetic db/db mouse^41^. We further evaluated whether fibrin matrices engineered with laminin-HBD peptides could enhance skin repair in a model of delayed wound healing, by controlling the release of VEGF-A165 and PDGF-BB in vivo. VEGF-A165 (100 ng/wound) and PDGF-BB (50 ng/wound) were co-delivered from the fibrin matrix onto full-thickness back-skin wounds in db/db mice, which provides a well-established and clinically relevant model of impaired wound healing^28^. Here, we particularly functionalized fibrin with the laminin peptide LAMA3_3043–3067_, since it bound to GFs and syndecans, and promoted fibroblast and endothelial cells adhesion in vitro (Figs. 4–6). Four groups were tested: fibrin only, fibrin functionalized with α_2_PI_1–8_-LAMA3_3043–3067_, fibrin containing GFs, and fibrin functionalized with α_2_PI_1–8_-LAMA3_3043–3067_ and containing GFs. Wound histology was analyzed after 4, 7, and 10 days, considering that wounds are normally fully closed after 15 days when treated with the fibrin matrix^28^. As a result, wounds that received fibrin matrices containing GFs or α_2_PI_1–8_-LAMA3_3043–3067_ peptide only did not differ from wounds treated with fibrin alone on day 7, neither in the amount of granulation tissue nor in the extent of wound closure (Fig. 8a–c). In contrast, the co-delivery of VEGF-A165 and PDGF-BB in fibrin functionalized with α_2_PI_1–8_-LAMA3_3043–3067_ led to a significantly faster wound closure after 7 days, as well as a significant increase in granulation tissue formation (Fig. 8a–c). GFs alone improved the amount of granulation tissue but not wound closure on day 10, suggesting that α_2_PI_1–8_-LAMA3_3043–3067_ peptide speeds the wound-healing process by these GFs. Representative wound morphology and images for all four treatments are presented in Fig. 8d and Supplementary Fig. 3. Clear differences in granulation tissue thickness and the extent of reepithelialization can be visualized when GFs were delivered within the α_2_PI_1–8_-LAMA3_3043–3067_ peptide-functionalized fibrin matrix compared to the other conditions.

Angiogenesis is a crucial step of wound healing in diabetic wounds, and both VEGF-A165 and PDGF-BB are angiogenesis inducers. We next examined endothelial cell proliferation (Fig. 8e and Supplementary Fig. 4). Co-delivery of VEGF-A165 and PDGF-BB in fibrin functionalized with α_2_PI_1–8_-LAMA3_3043–3067_ led to a significantly increased frequency of Ki67^+^, a proliferation marker, within CD31^+^CD45^−^ endothelial cells compared to other treatment groups on day 5. This is consistent with the increase in granulation tissue observed on day 7 as a result of delivery of GFs in fibrin functionalized with α_2_PI_1–8_-LAMA3_3043–3067_ (Fig. 8c).

Immune cells play a crucial role in wound-healing regulation^42^. We next examined the immune cell population in the wound in each treatment group (Fig. 8f, g and Supplementary Fig. 4). Delivery of GFs in fibrin functionalized with α_2_PI_1–8_-LAMA3_3043–3067_ slightly decreased the frequency of neutrophils within CD45^+^ cells compared to other treatment groups (Fig. 8f). On the other hand, delivery of GFs in fibrin functionalized with α_2_PI_1–8_-LAMA3_3043–3067_ increased the frequency of monocytes within CD45^+^ cells compared to other treatment groups (Fig. 8g). Among immune cells, neutrophils migrate first into wounds and then monocytes appear^43,44^. Therefore, this set of data suggests that delivery of GFs in fibrin functionalized with α_2_PI_1–8_-LAMA3_3043–3067_ promotes wound healing immunologically as well. Inclusion of α_2_PI_1–8_-LAMA3_3043–3067_ improved the GF delivery capacity of fibrin in vivo, resulting in an accelerated wound healing.

## Discussion

As a cell scaffold protein, laminin tightly regulates cell adhesion, motility, survival, and differentiation, thus playing a critical role in tissue homeostasis and wound healing^8^. In fact, although the expression of particular laminin isoforms depends on the tissue type and state, laminins reportedly promote tissue repair in muscle, nerve, liver, and skin^3,45,46^. In this study, we uncovered a novel property of laminin, showing that multiple laminin isoforms promiscuously bind to heparin-binding GFs with high affinities (Fig. 1). Although the binding between laminin and GFs has been mainly evaluated using immobilized proteins, we have also demonstrated that incorporation of laminin peptides prolongs GF retention in 3D fibrin matrices (Fig. 7).

Interactions between GFs and the ECM are known to be essential for controlling GF release kinetics in vivo, which strongly modulates tissue morphogenesis^28^. While sequestration of GFs within basement membranes was previously reported to be mediated through binding to glycosaminoglycans^47^, our data demonstrate that laminin can serve this GF reservoir function as well. In addition, the laminin diversity in different tissue ECMs and the different GF-binding profiles could be associated with regulation of GF contents and functions in specific tissues. For example, PDGF-BB has been reported to enhance muscle regeneration by activating satellite cell proliferation and migration^48^ and to protect the myocardium from ischemic injury^49^. Laminin-211 is the most abundant isoform in adult skeletal muscle tissue^50^ and is also expressed in the myocardium^51^. Therefore, the strong binding between PDGF-BB and laminin-211 suggests modulation of PDGF-BB action in muscle tissues through the interaction (Fig. 1).

Interestingly, most of the GFs that bind to laminin have also been shown to bind to other ECM proteins, such as fibrinogen and fibronectin, suggesting similar binding mechanisms^27,28,30,35^. However, a few exceptions can be noticed; for example, PDGF-CC showed specific binding to laminin, but neither to fibrinogen nor fibronectin^30,35^. In contrast, BMP-7, which binds specifically to fibronectin^27^ but not fibrinogen^30^, did not show binding to laminin. Moreover, kinetic analysis of laminin isoform-521 binding toward VEGF-A165, PDGF-BB, and PlGF-2 revealed *K*_D_ in nM range, highlighting the broad yet high affinity interactions between laminin and GFs.

Furthermore, we identified that GF binding to laminin mainly occurs at heparin-binding sites, by showing that heparin directly competes with GF–laminin interactions and dramatically reduces them when added at high concentration (Fig. 2). Additionally, all laminin HBDs tested in this study were able to bind to GFs (Figs. 3 and 4). Yet, unexpectedly, GF binding to laminin does not seem to be limited to HBDs, as a few non-heparin-binding peptides also bound to some GFs, notably LAMA3_3031–3043_ and LAMA5_3539–3550_. These peptides are human equivalents of reported mouse HBD peptides, called A3G75 and A5G94, respectively^32^. Although they do not show heparin binding under our experimental conditions, they may bind to GFs via another mechanism. Thus, the mechanism of GF-binding to laminin still remains incompletely clarified and may be resolved by further crystallography studies of GF–laminin complex.

Physiologically, proteolytic cleavage of LG4 and LG5 domains is crucial for the deposition of laminin in the native ECM^15,16,52^. Upon tissue injury, laminin is overexpressed, and LG4–LG5 domains accumulate in wounds^3,13^, wherein they promote tissue-healing mechanisms^53^. In this study, we particularly characterized laminin-derived peptides that are located just before the proteolytic cleavage site, in the linker between the LG3 and LG4 domains, or within the LG4–LG5 domains (Supplementary Table 1, Fig. 4a). On one side, we discovered three novel heparin-, GF-, and syndecan-binding peptides within the LG3–LG4 linker regions of LAMA3, LAMA4, and LAMA5, namely LAMA3_2932–2951_, LAMA4_1408–1434_, and LAMA5_3300–3330_, identifiable through their highly cationic sequences (Fig. 4). Since LAMA3, LAMA4, and LAMA5 are known to be predominantly present in their processed form (i.e., lacking LG4–LG5) in mature, unwounded skin^3,13–16^, it is likely that these peptides are exposed in vivo under homeostatic conditions, thus providing both GF ligands and cell adhesion sites in basement membranes. Interestingly, laminin LAMA1 is not proteolytically processed^14^, and LAMA2 does not contain such cationic sequences in the LG3–LG4 linker region, which might reflect functional differences between α-chain isoforms. On the other side, we identified five peptides in the LG4 and LG5 domains of LAMA3, LAMA4, and LAMA5 that displayed specific binding to GFs, in particular to VEGF-A165. Among them, LAMA3_3043–3067_, LAMA5_3539–3550_, and LAMA5_3417–3436_ additionally bound to PDGF-BB, FGF-2, and PlGF-2 with high affinities (Fig. 4). These GFs are well-known as key regulators of the wound-healing cascade, and are particularly involved in wound angiogenesis. Therefore, we propose that the reported positive effects of LG4–LG5 domains during wound healing might be related to promiscuous interactions with GFs, in addition to binding to syndecans and release of laminin-derived pro-angiogenic peptides^1,18,19^.

Previous studies have shown that the formation of ECM protein:GF complexes can synergistically enhance GF receptor signaling^28^. For example, simultaneous presentation of GFs and integrin-binding sites by an engineered fibronectin fragment (namely FN III9–10/12–14) incorporated into fibrin drastically improved the effect of VEGF-A165 and PDGF-BB on skin repair^28^. Here, we identified five laminin HBDs that are able to bind to both GFs and syndecan cell-surface receptors (Figs. 4 and 5), among which LAMA3_3043–3067_, LAMA4_1521–1543_, and LAMA5_3417–3446_ further promoted cell attachment (Fig. 6). Although syndecans are not known to directly activate major signaling pathways, they support cell adhesion and integrin signaling^54^. Moreover, direct binding of laminin peptides from LG domains to integrins has also been reported; for example, the integrin α3β1 binds to LAMA3_2932–2943_^55^. Nevertheless, in our assays, EDTA did not abolish cell adhesion, suggesting that initial cell attachment was mediated by syndecans rather than integrins (the binding of which is Ca^2+^ dependent). Consequently, and considering the short length of the laminin HBD peptides, it is unlikely that laminin HBD peptides can enhance GF signaling via synergy with integrins. In terms of the contribution of the cell adhesion properties of the laminin HBD to wound healing, we have previously shown that enhanced GF affinities against ECM proteins significantly promote skin wound healing^35^. Also, synthetic matrices incorporated with GFs and a GF-binding domain derived from fibrinogen, which does not contain a cell adhesion domain, promoted skin wound healing in our previous research^30^. Thus, we suppose that GF-binding properties, more than cell adhesion properties, of laminin HBDs in fibrin matrices substantially contribute to the promotion of wound healing.

Although GFs are promising drugs for tissue regeneration, their uncontrolled delivery upon application on wounded tissue has limited their clinical efficacy and safety to date^56,57^. For example, recombinant human VEGF-A has not been approved for clinical use by the U.S. Food and Drug Administration (FDA) due to a negative result in phase-II clinical trials^58^. PDGF-BB (Regranex in the clinic) has shown clinical efficacy, but safety issues such as cancer risk have been flagged, potentially due to high dosing^26,59^. Because 20 µg per wound of VEGF-A165 applied topically for five consecutive days were known to promote wound healing in the db/db mouse^40^ and 10 µg per wound of PDGF-BB did not significantly enhance wound healing^41^, we treated full-thickness back-skin wounds with a roughly 40- to 250-fold lower dose of GFs (combination of 100 ng of VEGF-A165 and 50 ng of PDGF-BB) delivered once in a fibrin matrix. Thus, controlling GF delivery to improve efficacy and dose reduction seems essential in future GF-based therapies and could be achieved by use of biomaterial matrices^28^.

Here, we showed that covalent incorporation of an engineered GF-binding domain derived from laminin, α_2_PI_1–8_-LAMA3_3043–3067_, into the fibrin matrix significantly enhanced the effect of VEGF-A165 and PDGF-BB on skin wound healing, by highly increasing GF retention into fibrin both in vitro and in vivo (Fig. 8). In contrast, wounds treated with the fibrin matrix containing GFs only, in which PDGF-BB and VEGF-A165 were not specifically retained in the fibrin matrices, had no detectable effect on wound healing at the tested dose (Fig. 8). Wounds treated with the fibrin matrix containing α_2_PI_1–8_-LAMA3_3043–3067_ only promoted wound closure slightly. This might be the result of trapping endogenous GFs. Considering the importance of angiogenesis in diabetic wounds and our observation of increased Ki67^+^ within CD31^+^CD45^−^ endothelial cells, the healing process induced by the fibrin matrix containing α_2_PI_1–8_-LAMA3_3043–3067_ and GFs was driven by enhanced angiogenesis in the wounds. Improved angiogenesis, which sustains the newly formed granulation tissue^60^, resulted from effective sequestration of VEGF-A165 and PDGF-BB (Fig. 7). Granulation tissue morphogenesis translated to improved morphogenesis at the level of the dermal epithelium, as reflected by faster wound closure.

We have previously shown that HBDs derived from fibrinogen (Fg β15^−^66_(2)_) and fibronectin (FN III9–10/12–14) promote wound healing in combination with GFs when incorporated into the synthetic matrix or fibrin matrix^28,30^. A main advantage of using the laminin HBD peptide for this purpose, compared to the previously described GF-binding domains, is production simplicity: the laminin HBD peptide is short enough to be chemically synthesized in large scale, rather than requiring recombinant expression. Furthermore, we showed that a laminin HBD can functionalize the fibrin matrix in both aspects as a GF reservoir and an adhesion-promoting cell scaffold (Figs. 6 and 7).

In conclusion, here, we show that multiple isoforms of laminin promiscuously bind GFs from the VEGF/PDGF, FGF, BMP, and NT families, in addition to HB-EGF and CXCL12γ, through their HBDs. By engineering a fibrin matrix displaying the LAMA3_3043–3067_ laminin HBD, as a demonstrative example, we show that the laminin HBD peptide promotes skin wound closure in the db/db mouse, as a model of delayed wound healing, when associated with VEGF-A165 and PDGF-BB. In addition to highlighting a GF-modulating function for laminin, an important tissue homeostasis and repair protein, we show that both GF- and cell-binding characteristics of a laminin HBD can promote tissue repair when incorporated within the fibrin matrix, which may be clinically useful.

## Methods

### GFs and chemokines

All GFs and chemokines were purchased in their mature forms, highly pure (>95% pure), carrier-free, and lyophilized^35^. VEGF-A121, VEGF-A165, PlGF-1, PlGF-2, PDGF-AA, PDGF-BB, PDGF-CC, PDGF-DD, FGF-1, FGF-2, FGF-6, FGF-7, FGF-9, FGF-10, FGF-18, BMP-2, BMP-3, BMP-4, BMP-7, β-NGF, NT-3, BDNF, IGF-1, IGF-2, HB-EGF, CXCL-11, and CXCL-12α were purchased from PeproTech. CXCL-12γ was purchased from R&D systems. Except for PDGF-DD and BMP-7, which were produced in eukaryotic cells, all GFs were produced in *Escherichia coli* and thus were not glycosylated. All GFs were reconstituted and stored according to the provider’s instructions to regain full activity and prevent loss of protein.

### Detection of laminin binding to recombinant GFs

Ninety-six-well ELISA plates (med-binding, Greiner Bio-One) were coated with 50 nM GFs in 100 µL/well of phosphate-buffered saline (PBS) at 37 °C for more than 2 h. After blocking with 2% BSA solution containing PBS and 0.05% Tween 20 (PBS-T), 10 nM recombinant human laminin isoforms (–111, –211, –332, –411, –421, –511, and –521) (>95% purity tested by SDS-PAGE, BioLamina) in 100 µL/well PBS were added. Bound laminin was detected with 100 µL/well rabbit anti-human LAMC1 polyclonal antibody (Assay Biotech, Catalog No. C13072, 1:1000 dilution in PBS) or rabbit anti-human LAMA3 polyclonal antibody (Assay Biotech, Catalog No. C13066, 1:1000 dilution in PBS). After incubation with biotinylated anti-rabbit antibody for 60 min at room temperature (RT), HRP-conjugated streptavidin (Jackson ImmunoResearch) was added. After 60 min of incubation at RT, 50 μL of TMB substrate (Sigma-Aldrich) was added. The reactions were stopped by adding 25 μL of 2 N H_2_SO_4_. Subsequently, the absorbance at 450 nm was measured with a reference of 570 nm.

### Production of recombinant laminin LAMA3_2928–3150_ protein

The sequence encoding for human *LAMA3* LG domain Ser2928-Cys3150 (linker domain and LG4 domain) was synthesized and subcloned into the mammalian expression vector pcDNA3.1(+) by Genscript. A sequence encoding for 6 His was added at the N-terminus for further purification of the recombinant protein. Suspension-adapted HEK-293F cells were routinely maintained in serum-free FreeStyle 293 Expression Medium (Gibco). On the day of transfection, cells were inoculated into fresh medium at a density of 1 × 10^6^ cells/mL. A total of 1 µg/mL plasmid DNA, 2 µg/mL linear 25-kDa polyethylenimine (Polysciences), and OptiPRO SFM media (4% final concentration, Thermo Fisher) were sequentially added. The culture flask was agitated by orbital shaking at 135 rpm at 37 °C in the presence of 5% CO_2_. Six days after transfection, the cell culture medium was collected by centrifugation and filtered through a 0.22-μm filter. Culture media was loaded into a HisTrap HP 5-mL column (GE Healthcare), using an ÄKTA pure 25 (GE Healthcare). After washing of the column with wash buffer (20 mM imidazole, 20 mM NaH_2_PO_4_, and 0.5 M NaCl, pH 7.4), protein was eluted with a gradient of 500 mM imidazole (in 20 mM NaH_2_PO_4_, 0.5 M NaCl, pH 7.4). The elusion solution was further purified with size-exclusion chromatography using a HiLoad Superdex 200PG column (GE Healthcare). All purification steps were carried out at 4 °C. The expression of laminin LG domain was determined by western blotting using anti-His tag antibody (BioLegend, Catalog No. 562504, 1:500 dilution) and the proteins were verified as >90% pure by SDS-PAGE.

### Surface plasmon resonance

SPR measurements were made with a Biacore 3000 SPR system (GE Healthcare). Laminin-521 or laminin LAMA3_2928–3150_ was immobilized via amine coupling on a C1 chip (GE Healthcare) for ~2000 or ~1000 resonance units (RU), respectively, according to the manufacturer’s instructions. VEGF-A165, PDGF-BB, or PlGF-2 was flown at increasing concentrations in the running buffer (0.01 M HEPES, pH 7.4, 0.15 M NaCl, and 0.005% v/v surfactant P20) at 20 μL/min. The sensor chip was regenerated with 50 mM NaOH for every cycle. Specific bindings of GFs to laminin were calculated by comparison to a non-functionalized channel used as a reference. Experimental results were fitted with Langmuir binding kinetics using BIAevaluation software (GE Healthcare).

### Inhibition of laminin–GF binding by heparin

ELISA plates (med-binding) were coated with 10 μg/mL laminin isoforms (–111, –211, –221, –411, –421, –511, and –521) in PBS for 2 h at 37 °C. Then, wells were blocked with 2% BSA-containing PBS-T and further incubated with 1 μg/mL each of VEGF-A165, PlGF-2, or FGF-2 for 60 min at RT with 10 μM heparin. Next, the wells were incubated with biotinylated anti-VEGF (R&D Systems, Catalog No. DY293B, 1:60 dilution), anti-PlGF (R&D Systems, Catalog No. DY264, 1:180 dilution), or anti-FGF-2 (R&D Systems, Catalog No. DY233, 1:60 dilution) antibodies. The antibodies were detected by streptavidin-HRP (R&D Systems). Signals were revealed and measured as described above.

### Detection of GF binding to laminin LG domain and laminin HBD

ELISA tests were performed as described above. In brief, ELISA plates were coated with 1 μg/mL of laminin alpha 3 LG domain recombinant protein, laminin alpha 4 LG domain recombinant protein (R&D Systems), laminin alpha 5 LG domain recombinant protein (LD BioPharma), or laminin peptide (sequences are described in Supplementary Table 1, chemically synthesized by Genscript) in PBS for 2 h at 37 °C. A concentration of 1 μg/mL of BSA served as non-binding protein control. After blocking with 2% BSA PBS-0.05% Tween 20 (PBS-T) solution, 1 μg/mL of the recombinant human proteins (VEGF-A121, VEGF-A165, PlGF-1, PlGF-2, PDGF-BB, or FGF-2) or 10 μg/mL of biotinylated heparin (Sigma-Aldrich) were added. Bound GF was detected with biotinylated antibodies for human VEGF-A, PlGF, PDGF-BB (R&D Systems, Catalog No. DY220, 1:180 dilution), or FGF-2 (R&D Systems). The antibodies were detected by streptavidin-HRP (R&D Systems). Signals were revealed and measured as described above.

### Detection of syndecan binding to laminin HBD

ELISA tests were performed as described above. In brief, ELISA plates were coated with 1 μg/mL laminin peptide (sequences are described in Supplementary Table 1, chemically synthesized by Genscript) in PBS for 2 h at 37 °C. A concentration of 1 μg/mL of BSA served as non-binding protein control. After blocking with 2% BSA PBS-T solution, 1 μg/mL of the recombinant human syndecan-1, syndecan-2, syndecan-3, and syndecan-4 (all syndecan proteins are histidine-tagged; SinoBiological) were added. Bound GF was detected with anti-histidine tag antibody (BioLegend, Catalog No. 562504, 1:1000 dilution). Signals were revealed and measured as described above.

### Cell adhesion assay

A total of 96-well plates (non-tissue culture treated, Greiner Bio-One) were pre-coated with 1 μg/mL with laminin HBD peptides in PBS for 2 h at 37 °C, followed by blocking with 2% BSA PBS for 1 h at RT. Cell adhesion assays were performed using human lung fibroblasts (Lonza) in FGM-2 medium (Lonza) or human umbilical vein endothelial cells (HUVEC; Lonza) in EGM-2 medium (Lonza) supplemented with 1% fetal bovine serum (FBS) and 100 µg/mL VEGF-A165, with or without 5 mM EDTA (Sigma-Aldrich). Cells were plated at 3000 cells/well on laminin peptide pre-coated plates and incubated for 30 min at 37 °C, 5% CO_2_. Then, the medium was removed, and wells were quickly washed three times with PBS. Cell numbers were quantified using a CyQUANT assay, according to the manufacturer’s instructions (Invitrogen). All cell lines were checked for mycoplasma contamination and used in passages from 5 to 8.

### Migration assay

A QCM 24-Well Colorimetric Cell Migration Assay Kit was used to perform migration assay. Both sides of inserts were coated with 0.1 μM of bovine collagen I (C4243, Sigma-Aldrich) for 1 h at 37 °C. Then, the inserts were washed with water, dried in a laminar flow cabinet, and disposed on 24-well cell culture plate covers. Solutions containing 30 ng/mL of VEGF-A165 preincubated with or without 0.1 μM of LAMA3_3043–3067_ peptide in a medium (MCDB-131, 0.05% BSA) were added to the bottom side of the transwell (500 μL/well). Directly thereafter, HUVEC cells (Lonza) in a medium containing 0.05% BSA (300 μL/transwell, 4 × 10^4^ cells/transwell) were added to the transwell upper parts. After 6 h, migrated cells were stained and absorbance at 560 nm was measured according to the manufacturer’s instructions.

### Release of GF from fibrin matrix

Fibrin matrices were generated with human fibrinogen (von Willebrand factor and fibronectin depleted, Enzyme Research Laboratories) as described previously^35^. In brief, fibrin matrices were generated with 8 mg/mL fibrinogen, 2 U/mL human thrombin (Sigma-Aldrich), 4 U/mL factor XIIIa (Fibrogammin; Behring), 5 mM calcium chloride (Sigma-Aldrich), 2 µM α_2_PI_1–8_-laminin peptide (sequences are described in Supplementary Table 1, chemically synthesized by Genscript), and 500 ng/mL recombinant human VEGF-A165 or PDGF-BB. Thus, the peptides were incorporated into the 3D fibrin matrix through enzymatic coupling, via the coagulation transglutaminase factor XIIIa, of the α_2_PI_1–8_ peptide sequence (NQEQVSPL) fused to the laminin peptide^28,61,62^. Fibrin matrix was polymerized at 37 °C for 1 h and transferred into 24-well Ultra Low Cluster plates (Corning) containing 500 μL of buffer (20 mM Tris-HCl, 150 mM NaCl, and 0.1% BSA; pH 7.4). A control well that served as a 100% released control contained only the GF in 500 μL of buffer. Every 24 h, buffers were removed, stored at −20 °C, and replaced with fresh buffer. For the 100% released control well, 20 μL of buffer was removed each day and stored at −20 °C. After 5 days, the cumulative release of GF was quantified by ELISA (DuoSet; R&D Systems), using the 100% released control as a reference.

### Retention of VEGF-A165 at the wound site

Ten C57BLKS/J-m/Lepr db (db/db) mice (The Jackson laboratory) aged 10–11 weeks were used. Their backs were shaved and four full-thickness punch-biopsy wounds (6 mm in diameter) were created in each mouse. Directly after, fibrin matrices [80 µL in total, fibrinogen (10 mg/mL), 2 U/mL human thrombin, 4 U/mL factor XIII, 5 mM calcium chloride, 2 µM α_2_PI_1–8_-LAMA3_3043–3067_, and 200 ng of recombinant human VEGF-A165] were polymerized on the wounds. To avoid drying of the matrices, the wounds were covered with non-adhering dressing (Adaptic, Johnson&Johnson), and then with adhesive film dressing (Hydrofilm, Hartmann). After 3 or 6 days, mice were sacrificed. The wounds were punched again, in order to recover the fibrinous matrices. Moreover, the tissue surrounding the wounds (2 mm beyond the wound margin) was removed. The tissue was transferred in 0.9 mL of tissue T-PER Tissue Protein Extraction Reagent (Thermo Scientific) containing 1 mg/mL of collagenase IV (Sigma-Aldrich), and homogenized with a tissue homogenizer. The tissue lysate was incubated for 1 h at 37 °C and 100 μL of a 5 M NaCl solution containing protease inhibitors (one tablet of protease inhibitor cocktail for 10 mL) was added to the lysate. The samples were centrifuged at 10,000 × *g* for 5 min, and the supernatants were stored at –80 °C. Recombinant human VEGF-A165 remaining in the fibrinous matrix and in the tissue surrounding the wound was quantified by ELISA (DuoSet, R&D Systems), using 200 ng of recombinant human VEGF-A165 as 100%. All animal experiments were performed with approval from the Veterinary Authority of the Institutional Animal Care and Use Committee of the University of Chicago.

### Mouse skin chronic wound-healing model

Twenty-seven 10–11-week-old C57BLKS/J-m/Lepr db (db/db) male mice (The Jackson Laboratory) were used. Their backs were shaved and four full-thickness punch-biopsy wounds (6 mm in diameter) were created in each mouse. Directly after, fibrin matrices [80 µL in total, fibrinogen (10 mg/mL), 2 U/mL human thrombin, 4 U/mL factor XIII, 5 mM calcium chloride, 2 µM α_2_PI_1–8_-LAMA3_3043–3067_, 100 ng of VEGF-A165, and 50 ng of PDGF-BB] were polymerized on the wounds. The wounds were covered with adhesive film dressing. Mice were single-caged after the wound surgery. After 4, 7, and 10 days, mice were euthanized and the skin wounds were carefully harvested for histological analysis.

### Histomorphometric analysis of wound tissue sections

An area of 8 mm in diameter, which includes the complete epithelial margins, was excised. Wounds were cut in the center into two and embedded into paraffin. Histological analysis was performed on 5-μm serial sections. Images were captured with an EVOS FL Auto microscope (Life Technologies). The extent of reepithelialization and granulation tissue formation was measured by histomorphometric analysis of tissue sections (H&E stain) using ImageJ software (National Institutes of Health). For analysis of reepithelialization, the distance that the epithelium had traveled across the wound was measured; the muscle edges of the panniculus carnosus were used as an indicator for the initial wound edges; and reepithelialization was calculated as the percentage of the distance of edges of the panniculus carnosus muscle. For granulation tissue quantification, the area covered by a highly cellular tissue was determined.

### Flow cytometric analysis of the wounds

Ten 10–11-week-old C57BLKS/J-m/Lepr db (db/db) male mice (The Jackson Laboratory) were used. Skin wounds were treated with fibrin matrices as described above. After 5 days, the wounded skins were removed as described above, cut into small pieces (<0.5 mm^2^) and transferred to 1 mL of an enzyme solution (collagenase D (1 mg/mL)), and agitated for 1 h at 37 °C. Then, the cells from digested wounds were resuspended in PBS, passed through a cell strainer, and centrifuged. Then, cells were stained for 15 min in 100 μL of 2% FBS in PBS-containing antibodies: anti-CD31 (MEC13.3, BD Biosciences, Catalog No. 563089, 1:200 dilution), anti-Ki67 (B56, BD Biosciences, Catalog No. 563756, 5 µL/test), anti-CD45 (30-F11, Catalog No. 103114, 1:400 dilution), anti-Ly6G (1A8, Catalog No. 127607, 1:200 dilution), anti-Ly6C (HK1.4, Catalog No. 128021, 1:200 dilution), and anti-CD11b (M1/70, Catalog No. 101227, 1:200 dilution). All antibodies were purchased from BioLegend if not otherwise described. Fixable live/dead cell discrimination was performed using Fixable Viability Dye eFluor 455 (eBioscience) according to the manufacturer’s instructions. Intracellular staining was performed using the Intracellular Staining Permeabilization Wash Buffer according to the manufacturer’s instructions (BioLegend). Cells were analyzed using a Fortessa (BD Biosciences) flow cytometer and FlowJo software (FlowJo, LLC). Gating strategies are shown in Supplementary Fig. 4.

### Statistical analysis

Statistical methods were not used to predetermine the necessary sample size, but sample sizes were chosen based on estimates from pilot experiments and previously published results such that appropriate statistical tests could yield significant results. Statistically significant differences between experimental groups were determined by one-way ANOVA followed by Tukey’s HSD post hoc test with Prism software (v7, GraphPad). Variance between groups was found to be similar by the Brown–Forsythe test. For nonparametric data (Fig. 6b, c), the Kruskal–Wallis test followed by Dunn’s multiple-comparison test was used. For ELISA data, the two-tailed Mann–Whitney *U* test was used. For the animal studies, experiments were not performed in a blinded fashion. Mice were randomized into treatment groups within a cage immediately before the wound surgery and treated in the same way. GF–laminin-binding ELISA assays were repeated four times. Wound-healing assays were repeated three times. *P* values less than 0.05 are considered to be significantly different. The *P* values less than 0.05 and 0.01 indicate symbols * and **, respectively.

### Data availability

The data that support the findings of this study are available from the authors upon reasonable request.

## Electronic supplementary material

Supplementary Infomation
