# Supplementary material for: Laminin heparin-binding peptides bind to several growth factors and enhance diabetic wound healing
Source: Nat Commun. 2018 Jun 4;9:2163. doi: 10.1038/s41467-018-04525-w (PMC5986797; doi:10.1038/s41467-018-04525-w)
Supplement: Supplementary file 1 — Supplementary Infomation [file 41467_2018_4525_MOESM1_ESM.docx]

**Supplementary Table 1. The sequences of laminin-derived peptides.**

| **Name (location) length** | **Peptide sequence** |
| --- | --- |
| LAMA3_2932-2951_ (Linker) 20 aa. | PPFLMLLKGSTRFNKTKTFR |
| LAMA3_3031-3043_ (LG4) 13 aa. | KNSFMALYLSKGR |
| LAMA3_3043-3067_ (LG4) 25 aa. | RLVFALGTDGKKLRIKSKEKCNDGK |
| LAMA4_1408-1434_ (Linker) 27 aa. | PLFLLHKKGKNLSKPKASQNKKGGKSK |
| LAMA4_1521-1543_ (LG4) 23 aa. | TLFLAHGRLVYMFNVGHKKLKIR |
| LAMA5_3300-3330_ (Linker) 31 aa. | TPGLGPRGLQATARKASRRSRQPARHPACML |
| LAMA5_3312-3325_ (Linker) 14 aa. | ARKASRRSRQPARH |
| LAMA5_3417-3436_ (LG4) 20 aa. | RQRSRPGRWHKVSVRWEKNR |
| LAMA5_3539-3550_ (LG5) 12 aa. | TLPDVGLELEVR |
| LAMA3_3043-3067_ Scr1 25 aa. | RLVKALKTDKFLGRIGSEKCNDKGK |
| LAMA3_3043-3067_ Scr2 25 aa. | RKTDALELVFLKKGGIGSKKCNDKR |
| LAMA3_3043-3067_ Scr3 25 aa. | CRKKKRKKKALLLGIGDFNSEVTDG |
| LAMA3_3043-3067_ Scr4 25 aa. | KKRKLVALTDFLGICGSENDGRKKK |
| LAMA3_3043-3067_ Scr5 25 aa. | LVRAKLTDKFLGKRIGSKECNKDKG |
| LAMA3_3043-3067_ Scr6 25 aa. | ALLLGIGRDFNKKKRKKKSEVTDGC |
| α_2_PI_1-8_-LAMA3_3043-3067_ 33 aa. | NQEQVSPLRLVFALGTDGKKLRIKSKEKCNDGK |
| α_2_PI_1-8_-LAMA5_3312-3325_ 22 aa. | NQEQVSPLARKASRRSRQPARH |

**Supplementary Table 2. Summary of laminin-derived peptide interactions.**

++ indicates high affinities, + indicates medium/low affinities. The laminin-derived peptide tested *in vivo* is highlighted in gray.

| **Laminin-derived  peptides** | Interaction with | | | Cell adhesion | |
| --- | --- | --- | --- | --- | --- |
|  | **Heparin** | **GF** | **Syndecan** | **Fibroblast** | **HUVEC** |
| LAMA3_2932-2951_ | ++ | + | + | + | + |
| LAMA3_3031-3043_ |  | + | + | + |  |
| **LAMA3_3043-3067_** | ++ | ++ | ++ | ++ | ++ |
| LAMA4_1408-1434_ | ++ | ++ | ++ |  |  |
| LAMA4_1521-1543_ | ++ | + | ++ | + | + |
| LAMA5_3300-3330_ | ++ | + | ++ |  |  |
| LAMA5_3312-3325_ |  |  | + |  |  |
| LAMA5_3417-3436_ | ++ | ++ | ++ | + |  |
| LAMA5_3539-3550_ |  | + |  |  |  |

**Supplementary Fig. 1. Scrambling the laminin HBD sequence decreases the GF binding capacity**

Affinity of GFs against chemically synthesized peptides that are scrambled (Scr) the sequence of LAMA3_3043-3062_. ELISA plates were coated with 10 µg/mL laminin peptide and further incubated with VEGF-A165, PlGF-2, PDGF-BB, or FGF-2. Concentrations were 1 μg/mL for GFs. Bound GF was detected with a specific antibody for each GF (n = 4, mean ± SEM). Statistical analyses were done using one-way ANOVA. *p < 0.05, **p < 0.01. Sequence of the peptides are described in Table 2. Two experimental replicates.

**Supplementary Fig. 2. Laminin HBD did not enhance the endothelial cells migration**

4×10^4^ HUVEC cells were added to the transwell upper parts. Solutions containing 30 ng/mL of VEGF-A165 preincubated with or without 0.1 μM of LAMA3_3043-3067_ peptide were added to the bottom side of the transwell. The signals of the cells that passed through a migration transwell after 6 hrs of incubation were measured. (means ± SEM, n = 4). Statistical analyses were done using one-way ANOVA. **P < 0.01. Two experimental replicates.


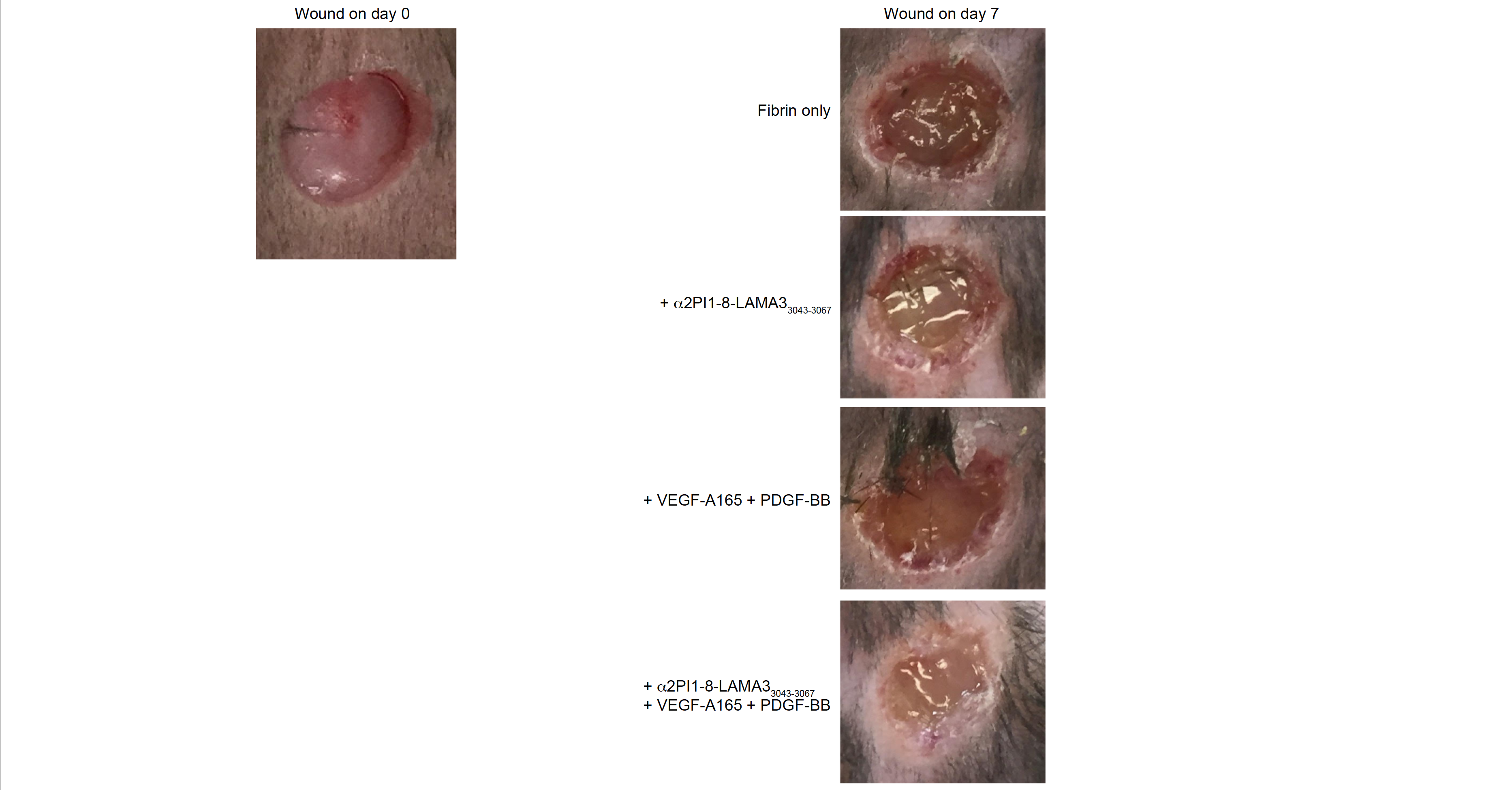


**Supplementary Fig. 3. Photos of the wounds**

Full-thickness back-skin wounds were treated with combined VEGF-A165 (100 ng/wound) and PDGF-BB (50 ng/wound). Four groups were tested: fibrin only, fibrin functionalized with α_2_PI_1–8_-LAMA3_3043-3067_ peptide, fibrin containing admixed GFs, and fibrin functionalized with α_2_PI_1–8_-LAMA3_3043-3067_ peptide containing GFs. Representative pictures of wounds after 0 and 7 days are presented.

** Supplementary Fig. 4. Gating strategy for flow cytometric analysis**

Flow cytometric gating strategies showing (A) proliferation of CD31^+^CD45^-^Ter119^-^ endothelial cells assessed by Ki67^+^ marker cells (Ki67^+^ of CD31^+^CD45^-^Ter119^-^ cells pre-gated on live cells) and (B) Ly6G^+^CD11b^+^ neutrophils of CD45^+^ cells and Ly6C^+^CD11b^+^ monocytes of CD45^+^ cells, pre-gated on live cells.
